# Adult Embryonal Sarcoma of the Liver: Management of a Massive Liver Tumor

**DOI:** 10.1155/2016/5625762

**Published:** 2016-11-08

**Authors:** Daniela Treitl, Alexandra Roudenko, Siba El Hussein, Magda Rizer, Philip Bao

**Affiliations:** ^1^Mount Sinai Medical Center, 4300 Alton Road, Miami Beach, FL 33140, USA; ^2^Mount Sinai West, 1000 10th Avenue, New York, NY 10019, USA

## Abstract

Undifferentiated embryonal sarcomas of the liver are extremely rare cases in adults. We report the case of a 30-year-old male who presented with early satiety and abdominal pain due to a massive tumor originating from the left liver and occupying the entire epigastrium. The patient underwent bland embolization in an attempt to decrease the size of the tumor. He then underwent a formal left hepatectomy with resection of liver segments 2, 3, and 4. Extrahepatic inflow control of the portal vein and hepatic artery was performed prior to parenchymal transection. No Pringle maneuver was required. Pathology analysis showed a 45 cm tumor consistent with an undifferentiated embryonal sarcoma and negative microscopic margins. The epidemiology, treatment, and prognosis of this unusual cancer presentation are reviewed.

## 1. Introduction

Undifferentiated embryonal sarcoma (UES) of the liver is typically an aggressive childhood tumor with poor prognosis. The first review of this pathology was done by Stocker and Ishak in 1978, where it was found that most cases were in children six to ten years of age with no predilection for gender and involving the right lobe in 90% of the cases [1].

Although this rare tumor predominantly affects children, it has been reported in adults [2, 3]. Unlike pediatric cases, one review noted UES in adults as more commonly observed in females, still in the right lobe of the liver, and with the mean age at diagnosis of 51 [4]. Curative intent surgical resection is the treatment of choice although systemic chemotherapy and regional techniques such as transcatheter arterial chemoembolization (TACE) may have benefit [4, 5]. In addition, orthotopic liver transplantation has been used in unresectable cases to achieve local control in the pediatric population [6, 7]. We present a case of a massive UES of the liver in an adult male, highlighting the clinical presentation, pathological findings, and surgical management.

## 2. Case Presentation

**Figure 1:**
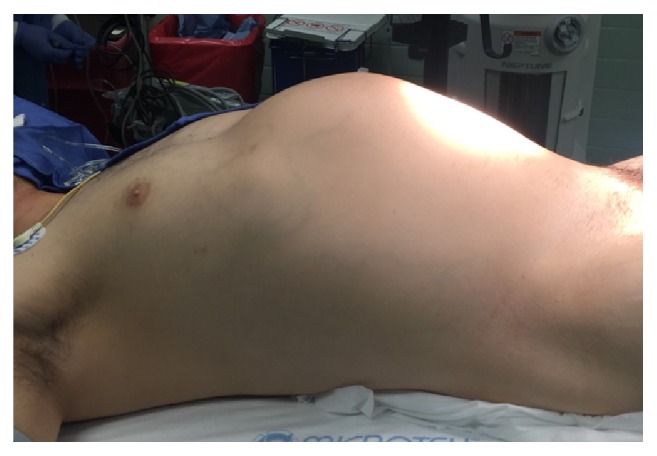
Abdominal mass on physical exam.

A 30-year-old male with no prior medical history presented with nonradiating upper abdominal pressure and pain associated with early satiety and bloating. This was progressive over approximately 2 months before he sought medical attention. His physical exam demonstrated an easily visualized and palpable firm epigastric mass (Figure 1). There were no signs of chronic liver disease such as ascites or jaundice.

Standard tumor biomarkers such as CEA, AFP, and CA19-9 were normal, as was serum bloodwork for liver metabolic and synthetic function. Subsequent imaging workup demonstrated a large heterogeneous liver tumor approximately 27 × 13 × 20 cm in size, occupying his entire upper abdomen with mass effect and posterior displacement of the pancreas and stomach.

**Figure 2:**
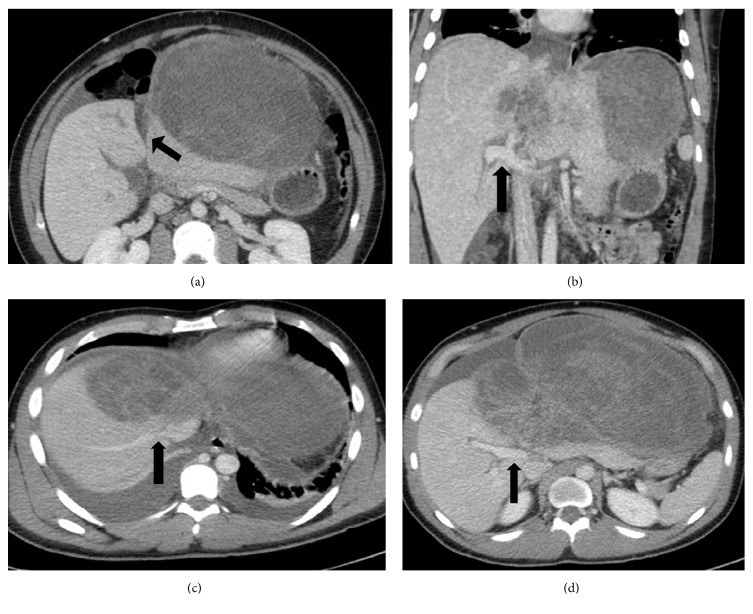
CT abdomen and pelvis with intravenous contrast showing large, heterogeneous left hepatic mass. Characterization of mass with regard to hepatic landmarks. (a) Arrow points to umbilical fissure. (b) Arrow identifies portal vein bifurcation. (c) Arrow points to juncture of right hepatic vein with inferior vena cava. (d) Arrow indicates right portal vein.

Multiphase contrast enhanced computed tomography (CT) scan showed the tumor originating from the left liver spanning segments 2, 3, and 4 with no evidence of biliary obstruction. However, the left portal vein was not well-visualized beyond the portal bifurcation suggesting severe compression or occlusion. Similarly, the middle and left hepatic veins were not seen (Figure 2). There were felt to be reactive ascites and a right pleural effusion but no clear evidence for metastatic disease in the thorax or abdomen.

Percutaneous biopsy showed spindle cells consistent with an undifferentiated sarcoma. After multidisciplinary review, the patient was considered a surgical candidate but preoperative downsizing might help from a technical standpoint. Tumor embolization was thought to be feasible and worth attempting to achieve this. The patient thus underwent bland embolization to the tumor via the left hepatic artery. Approximately two weeks after procedure, CT angiogram showed unfortunately no significant change in size although perhaps with some tumor necrosis related to the procedure.

This mass continued to be highly symptomatic and it was felt that further surveillance would result in being minimal if any shrinkage of the tumor; thus resection for both palliation and potential cure was attempted. Given normal preoperative liver function, it was felt that the anticipated liver remnant after left hepatectomy would be sufficient with little risk for posthepatectomy liver failure.

**Figure 3:**
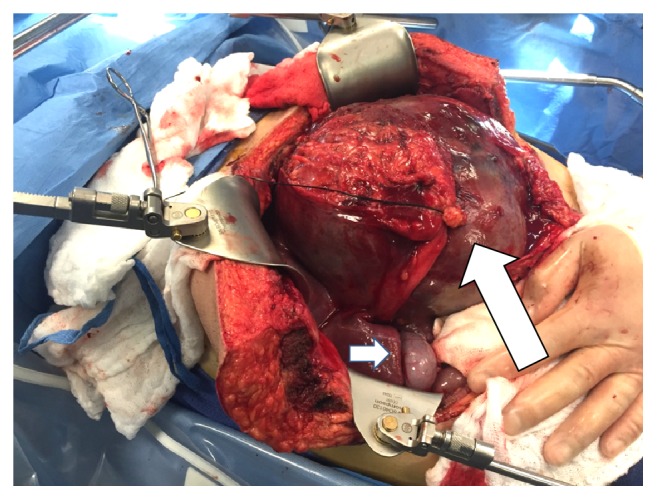
Tumor occupying entire epigastrium. Large arrow shows the round ligament secured by a suture. Short arrow points to gallbladder.

A chevron incision with midline extension allowed sufficient exposure of the porta hepatis and suprahepatic vena cava. There were extensive inflammatory changes around the tumor which appeared necrotic and there were hemorrhagic appearing ascites. The omentum was broadly adherent to the left side of the tumor, perhaps at the prior biopsy site. The tumor appeared to originate in segment 2 or 4 of the liver and essentially had replaced the entire left liver occupying the left upper quadrant and epigastrium (Figure 3). It was compressing the anterior aspect of the stomach as well as the spleen laterally, and separating these structures required ligation of the greater curvature gastroepiploic vessels.

**Figure 4:**
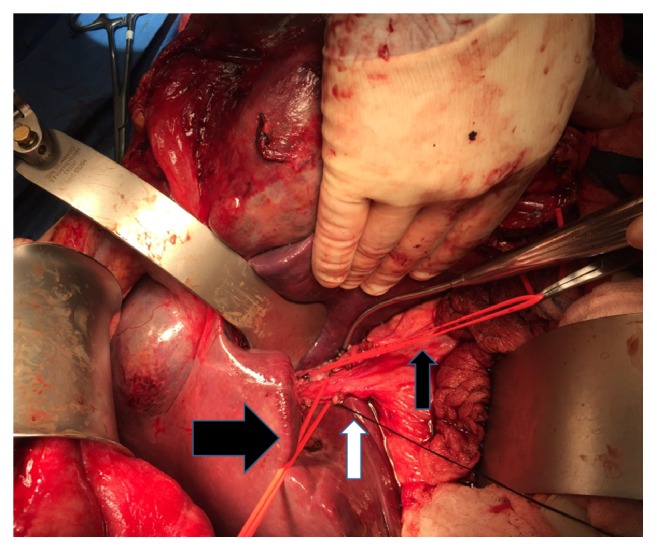
Small black arrow indicates vessel loop around the left portal vein. Large black arrow indicates loop around common hepatic bile duct. White arrow shows suture on cystic duct stump.

The bulk of the tumor did not permit rotation of the liver about the cava and thus no significant mobilization was attempted in favor of an anterior approach to the liver transection. Thus the transverse colon was retracted down and the porta hepatis exposed by gently elevating the front edge of the liver and working upwards along the hepatoduodenal ligament. Cholecystectomy was performed followed by exposure of the common bile duct and hepatic arteries. The left hepatic artery was divided at its origin. The portal vein was dissected to the umbilical fissure where the left portal vein branch was identified. There was insufficient space to divide the vessel but a silicone loop could encircle it to occlude the portal inflow to the left liver (Figure 4). With this selective inflow control to the left liver, it was felt that a formal Pringle maneuver would not then be required during liver transection. The hilar plate was lowered and the left bile duct was identified just to the right of the umbilical fissure.

Superiorly, dissection was carried back to the anterior cava and the groove between the right hepatic vein and the middle hepatic vein was identified and developed. An attempt at a hanging maneuver to pass a traction tape between the vena cava and liver was unsuccessful due to anterior compression of the cava at segment 4. Intraoperative ultrasound was performed to confirm the location of the right portal vein as well as the middle hepatic vein. The middle hepatic vein was severely compressed laterally by the tumor with no clear plane between the two. Segment 4B of the liver was more or less free of tumor and that was where the parenchymal transection was initiated using a crush and clip technique as well as harmonic scalpel, marching down the quadrate lobe towards the left bile duct. Transection was continued through segment 4B towards segment 4A, and the left bile duct was divided sharply.

The liver could now be rotated to expose the upper aspect of the ligamentum venosum which was divided. To provide a gross margin of approximately 1 cm, the line of parenchymal transection was extended into segment 8, crossing to the right of the upper middle hepatic vein. The left and middle hepatic veins were then isolated and divided using a vascular stapler where they joined the inferior vena cava. A plane was developed along the anterior cava and the parenchymal transection completed using the harmonic scalpel. The left bile duct stump was closed primarily and completion ultrasound confirmed inflow to the right liver remnant. Additional hemostasis was achieved using a bipolar sealer and a right chest tube was placed to drain any anticipated reactive pleural effusion. Estimated blood loss for the procedure was 1,500 cc and operative time was 440 minutes. He received no transfusions during the case but was given 2 units on postoperative day 3 for symptomatic anemia. No peritoneal drain was left. The patient's hospital course was complicated by ileus and ascites leak from the apex of the incision which resolved with conservative medical management, and he was discharged home by POD#12. At three-month follow-up, the patient is doing well and has returned to work. His oncologist initiated doxorubicin and ifosfamide for adjuvant therapy and he will get surveillance imaging every 3 months.

## 3. Pathology

**Figure 5:**
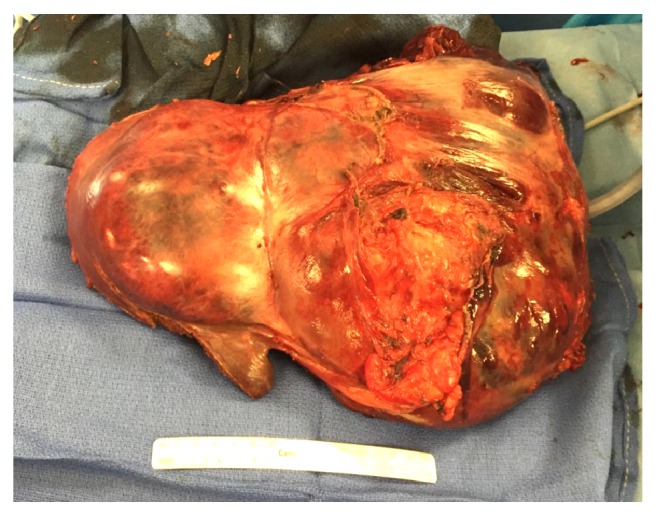
Resection specimen.

Gross examination revealed a 45 × 20 × 10 cm specimen with a dark red irregular nodular contour (Figure 5).

**Figure 6:**
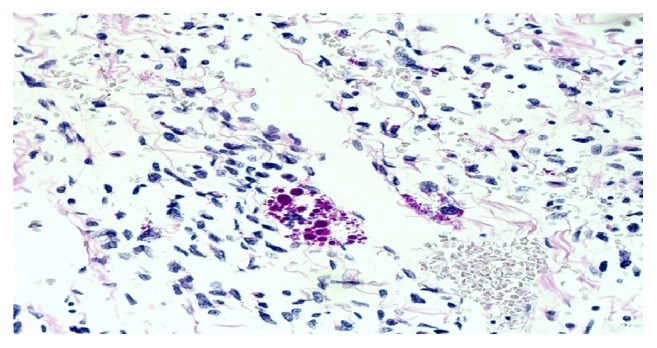
PAS positive hyaline globules (high power, H&E).

Microscopic examination revealed a malignant neoplasm composed predominantly of spindle cells and pleomorphic giant cells in a myxoid background. The margins were free of tumor. The tumor showed high cellularity with perivascular accentuation. Many of the pleomorphic giant cells showed numerous eosinophilic intracytoplasmic globules, which were PAS positive and diastase resistant (Figure 6).

**Figure 7:**
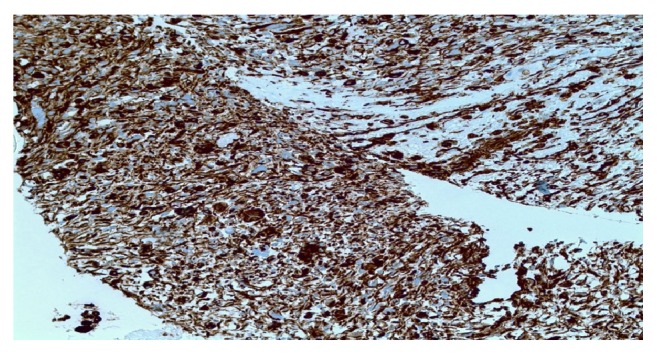
Vimentin positive stain (high power, H&E).

Mitotic figures were easily identified, most of which were atypical. Extensive hemorrhage and areas of necrosis were present. A component of the tumor was seen in association with cystic spaces lined by biliary-type epithelium. The neoplastic cells were negative for AE1/AE3, CD117, myogenin, and CD34. Vimentin stain was positive (Figure 7).

**Figure 8:**
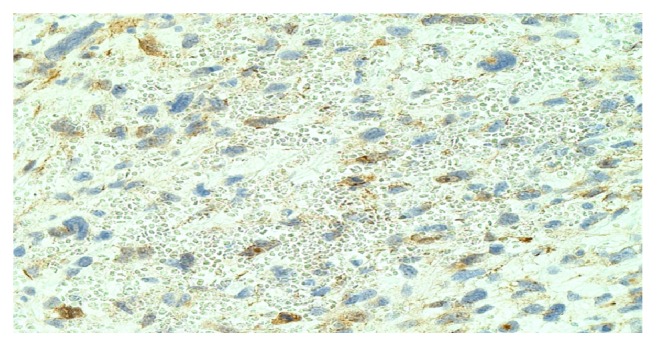
Beta-catenin showing predominantly a cytoplasmic pattern of staining in the tumor cells, with scattered nuclei showing mild to moderate immunoreactivity (high power, H&E).

A subpopulation of tumor cells was positive for Desmin. Few cells were positive for CD31. Immunohistochemistry for beta-catenin showed predominantly a cytoplasmic pattern of staining in the tumor cells, with scattered nuclei showing mild to moderate immunoreactivity (Figure 8).

This immunoprofile together with the morphology was consistent with a diagnosis of undifferentiated embryonal sarcoma of the liver.

## 4. Discussion

UES of the liver are rare and highly malignant childhood tumors, with few cases reported in adults, more commonly found in adult females and in the right lobe [4]. One review of all reported cases of patients diagnosed with UES who were at least 15 years of age or older consisted of 67 patients and found a slight female predominance (57%) [3]. Others report similar figures such as a review of 24 patients with a female predominance of 62.5% in patients aged ≥30 years with UES [4].

Radiographically, UES of the liver tend to present as large, usually solitary masses with imaging features often suggestive of a cystic lesion, secondary to the high water content in the myxoid stroma [8–10]. This potentially can lead to delays in diagnosis if mistaken for a benign cystic lesion. Solid appearance is most reliably demonstrated on ultrasound [8, 10]. Such discordant imaging findings of a predominantly solid lesion on ultrasound and cystic lesion on CT are felt to be highly suggestive of UES of the liver [11].

Cross-sectional imaging with multiphase contrast CT or magnetic resonance imaging (MRI) usually reveals a large hypodense mass, often well circumscribed with a pseudocapsule, multiple hyperdense septations of various thickness, and predominantly water attenuation with rare calcifications [9–11]. Delayed contrast enhanced imaging may be helpful in clarifying the solid nature of the tumor although there is usually a lack of or just mild, heterogeneous, and peripheral enhancement secondary to the extensive central necrosis and/or cystic change [10–13]. On angiography, these lesions are most often hypovascular; however, avascular and hypervascular appearances have been reported [9, 10]. Intratumoral hemorrhage may be seen and usually is best appreciated on MRI [13]. PET/CT has been reported to be useful in evaluating response to chemotherapy treatment [9].

UES of the liver has generally been associated with a poor prognosis with median survival of 29 months and 1- and 2-year survival of 61% and 55%, respectively [3]. As with most sarcomas, regardless of histogenesis, complete surgical resection is the mainstay of treatment when technically feasible. However, significantly better survival has been observed for patients receiving adjuvant chemotherapy as opposed to surgery alone [3, 4]. In one study, 42% of patients undergoing complete resection without adjuvant therapy recurred at 8 months compared to 23% at 28 months [3]. Chemotherapy regimens reported in the literature have included vincristine, actinomycin, ifosfamide, doxorubicin, cisplatin, and etoposide [3, 14]. It appears that the best treatment strategy for patients with UES is complete surgical resection and adjuvant chemotherapy [3, 14]. There is little published experience regarding the use of radiation therapy in UES, but the addition of radiation therapy may improve survival especially in patients who cannot undergo complete surgical resection [14, 15].

Recently, interventional radiology techniques are being considered in an attempt to decrease the toxicity of chemotherapy and decrease tumor size preoperatively. A report of two pediatric cases treated with TACE prior to resection reported a 23–31% decrease in tumor size [16]. In general, embolization techniques have been successful in the treatment of liver lesions with a response rate of 12% by RECIST criteria reported for transarterial embolization (TAE) and TACE independent of pretreatment lesion size and vascularity with a greater benefit seen with the addition of chemotherapy compared to bland embolization [17]. Liver transplantation has also been undertaken for UES of the liver in selected pediatric patients, showing good long term results particularly when patients are unable to have complete surgical resection [6, 7]. In the current case, had the tumor been unresectable, there may have been a role for transplantation. It should be noted, however, that to our knowledge this would have been the first use of transplant for an adult with this type of cancer.

As for the technical aspects of an operation for large or poorly located liver tumors, preoperative assessment of an adequately sized and functioning future liver remnant (FLR) may require formal liver volumetry. An FLR of at least 20–25% in healthy liver is needed and may be augmented by preoperative portal vein embolization of the side to be resected. During surgery, the surgeon must be prepared to employ a number of techniques particularly for minimizing hemorrhage. Total vascular isolation with infra- and suprahepatic vena cava clamping with or without venovenous bypass is an option. Low central-venous pressure anesthesia and the capability for rapid transfusion are also important. Other more radical techniques such as ex situ liver resection and hepatic autotransplantation have also been described [18].

In summary, adult embryonal sarcoma of the liver is a rare neoplasm that should be considered in the differential diagnosis of liver lesions, with complete surgical resection the treatment of choice. Given its poor prognosis, multimodality interventions are quite reasonable to optimize surgical results and decrease recurrence rates, but there is no clear standard of care outside resection as these tumors are so rare, limiting the possibility of prospective study. The case presented here gives a basis for the approach to resections of massive liver lesions including the use of anterior approach and selective inflow control. Long-term follow-up and additional multiple institution reviews are needed to formulate best practice strategies for this rare tumor.
